# 3D-Printing of Silk Nanofibrils Reinforced Alginate for Soft Tissue Engineering

**DOI:** 10.3390/pharmaceutics15030763

**Published:** 2023-02-24

**Authors:** Zahra Mohammadpour, Mahshid Kharaziha, Ali Zarrabi

**Affiliations:** 1 Department of Materials Engineering, Isfahan University of Technology, Isfahan 84156-83111, Iran; 2 Department of Biomedical Engineering, Faculty of Engineering & Natural Sciences, Istinye University, Istanbul 34396, Turkey

**Keywords:** hybrid hydrogel, silk nanofibril, alginate, three-dimensional printing, mechanical performances, rheological properties

## Abstract

The main challenge of extrusion 3D bioprinting is the development of bioinks with the desired rheological and mechanical performance and biocompatibility to create complex and patient-specific scaffolds in a repeatable and accurate manner. This study aims to introduce non-synthetic bioinks based on alginate (Alg) incorporated with various concentrations of silk nanofibrils (SNF, 1, 2, and 3 wt.%) and optimize their properties for soft tissue engineering. Alg-SNF inks demonstrated a high degree of shear-thinning with reversible stress softening behavior contributing to extrusion in pre-designed shapes. In addition, our results confirmed the good interaction between SNFs and alginate matrix resulted in significantly improved mechanical and biological characteristics and controlled degradation rate. Noticeably, the addition of 2 wt.% SNF improved the compressive strength (2.2 times), tensile strength (5 times), and elastic modulus (3 times) of alginate. In addition, reinforcing 3D-printed alginate with 2 wt.% SNF resulted in increased cell viability (1.5 times) and proliferation (5.6 times) after 5 days of culturing. In summary, our study highlights the favorable rheological and mechanical performances, degradation rate, swelling, and biocompatibility of Alg-2SNF ink containing 2 wt.% SNF for extrusion-based bioprinting.

## 1. Introduction

Tissue engineering (TE) has been applied to develop tissue and organ substitutes for clinical transplantation and restore organ function [1,2,3]. In this strategy, the scaffold made of biocompatible materials supporting cell functions is the crucial component. Currently, three-dimensional (3D)-bioprinting is known as an emerging strategy for creating scaffolds to link the divergence between artificially engineered structures and native tissues [4,5]. The unique advantage of this strategy is the fabrication of 3D functional and predesigned constructs by a precise layer-by-layer deposition of various types of appropriate materials, called inks and living cells, to regenerate target tissues. Moreover, the ability of 3D bioprinting for various cells in an orderly manner mimicking heterogeneous buildings of native tissues is another advantage of this strategy [6].

The preparation of inks is directly related to the working mode of 3D printing, including droplet-based, extrusion-based, laser-induced forward transfer, and stereo lithography techniques. Extrusion-based bioprinting is one of the interesting explored techniques that extrudes continuous fibers to form 3D scaffolds [7]. It shows promising properties, including versatility, multiple modes of solidification, and the ability to print complex structures [6]. In this system, inks should be well extrudable to avoid obstruction during the process. In addition, it should generate 3D structures with the desired profile reliability and mechanical solidity [8]. Accordingly, inks often need additional upgrading in printability, mechanical support, and bioactivity [9]. In this regard, applying inks with shear-thinning and thixotropic characteristics to decrease the force essential to motivate ink extrusion is promising. Shear-thinning permits inks to be placed via lower extrusion forces, whereas thixotropy could protect the shape of 3D-printed constructions before further crosslinking [10].

Various types of natural polymers have been applied in extrusion-based 3D printing approaches thanks to their promising biocompatibility and adjustable mechanical robustness. Among various types of hydrogels, alginate, a polysaccharide derived from different kinds of brown seaweed, is considered a suitable ink in many bioprinting techniques, given the distinctive properties, including biocompatibility, shear-thinning, biodegradability, ease of processing and fast gelation [11]. However, alginate presents challenges of mechanical instability and high fluidity, which is unfavorable for good shape fidelity [12]. Moreover, 3D-printed alginate hydrogels could not provide satisfactory mechanical performances and often show high volume shrinkage after ionic crosslinking. These issues could be overcome by various crosslinking strategies, surface coating, and composite formation [12,13]. The formation of hybrid inks based on alginate is one of the main strategies which has been widely developed [12]. Aartad et al. [14] studied the effect of mechanically-fibrillated and oxidized cellulose nanofibrils (CNFs) on the mechanical and rheological performances of 3D-printed alginate. It was concluded that alginate-CNF hydrogels revealed greater Young’s modulus and weaker syneresis than pure alginate making it more suitable for tissue engineering. Marksted et al. [15] also reported the bioink of alginate –CNFs and found that the addition of CNFs induced shear-thinning properties to the alginate ink leading to high shape fidelity and increased printing resolution.

Silk fibroin (SF), extracted from silkworms with unique properties of low immunogenicity, tunable mechanical performances, biodegradation, and biocompatibility [16], could also be considered for the engineering of bone [17], cartilage [18], and artery [19] and is used in both natural fiber and regenerated forms [18,20]. SF could also be simply administered into hydrogels offering a chance to be applied as bioinks in bioprinting [21]. Gelation of SF is prompted via the structural transition from the random coil to a β-sheet [22]. However, poor crosslinking and variation of viscosity are the barriers hindering the printing of pure SF hydrogel. To overcome these issues, 3D-printed alginate-silk structures have been reported in previous studies. For instance, Joshi et al. [23] reported alginate-gelatin bioink loaded with silk fibers for osteochondral grafts. In that study, alginate was used as a modifier to create printable ink. Aharonov et al. [24] also presented laminate constructed from long natural silk and fibroin fibers embedded in an alginate hydrogel matrix. In the mentioned study, different fiber volume fractions were characterized to tailor-design their mechanical behavior. To improve the mechanical properties of SF hydrogels, such as tensile strength and transparency, silk nanofibrils (SNFs) have also been proposed. SNFs as a natural protein biomaterial platform with diameters of 20–100 nm are the basic mesoscopic structural units of the hierarchical structure of silk materials [25]. SNFs not only have the benefits of availability, low price, biocompatibility, and degradability, but they could also significantly promote the mechanical toughness and biological functions of silk components [26,27,28]. These properties make SNFs attractive for various tissue engineering applications [29,30]. Moreover, contrary to silk, SNF may have a significant effect on the viscoelastic responses of inks. In order to develop 3D-printed structures with exact and precise shapes, accurately controlled pore structures, distinctive mechanical characteristics, and supporting cells, the formation of inks with viscoelastic properties is crucial [30]. Similar to other nanofibrous structures, SNF can be utilized as a rheological modifier for the inks, supporting the viscoelastic response essential for 3D printing. SNF exhibits shear thinning behavior, which facilitates the extrusion of inks. At the same time, it could keep self-supporting and high shape fidelity [21]. In addition, the incorporation of SNF into inks provides structural similarity of 3D-printed scaffolds to ECMs possessing filamentous architecture, consequence to the promotion of cell growth [31]. Therefore, the use of SNF instead of silk with micro-scale fibers in alginate matrix, might be promising to improve the viscoelastic responses, interaction of the matrix and the nanofibrils and cell responses.

Accordingly, this study aims to combine the fast gelation properties of alginate with the mechanical properties of SNFs to develop hybrid ink containing alginate–SNFs for soft tissue engineering. It is hypothesized that SF in nanofibril morphology and large matrix-nanofibril interfacial have critical effects on the mechanical and shear-thinning properties (printability) and cell responses of 3D-printed alginate.

## 2. Materials and Methods

### 2.1. Materials

Alginic acid sodium salt from brown algae (Mw = 80,000–120,000 g·mol^−1^, mannuronic/guluronic ratio of 1.56), CaCl_2_, LiBr, and NaHCO_3_ were purchased from Merck Co. (Germany). High-quality raw cocoon of silkworm was purchased from Noghan company (Isfahan, Iran). Dialysis membrane (cut-off ~12–14 kDa) was obtained from Betagen Co., Mashhad, Iran. Moreover, double distilled water (DDW) was used in all experiments.

### 2.2. Synthesis of Silk Nanofibrils (SNFs)

**Figure 1:**
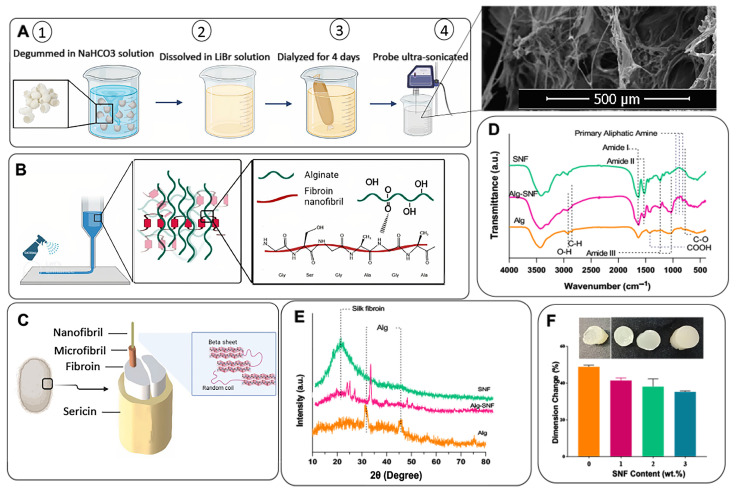
Schematic outlining (**A**) SNF synthesis process (Inset: FESEM image of the SNFs), (**B**) 3D printing strategy, and Alg-SNF interaction. (**C**) Nanofibril structure of silk fibroin consisting of random coils and β-sheet structures extracted from the silk cocoon. (**D**) FTIR spectra of Alginate, SNF, and Alg-SNF hydrogel. (**E**) XRD patterns of Alginate, SNF, and Alg-SNF hydrogel. (**F**) Diameter change of the Alg-SNF hydrogels after cross-linking. Photographs of hydrogels after cross-linking, showing the significantly different diameters after the crosslinking process.

The SNF extraction process is schematically described in Figure 1A. Silk was first degummed using a 0.02 M sodium hydrogen carbonate solution to produce pure fibroin by removing the sericin covering silkworm cocoons, according to previous studies [32]. Consequently, SNF was extracted from SF by dissolving it in 9.3 M LiBr solution for 2 h, according to previous protocols with minor modifications [33]. To eliminate LiBr, the aqueous SNF solution was dialyzed against DDW for 4 days while DDW was continuously stirred and replaced with fresh DDW every day. Before ink formation, the SNF solution was subjected to ultrasound treatment for 20 min using probe sonication equipment (Fisher Sonic Dismembrator, USA) to enhance random coil to β-sheet transition [34].

### 2.3. Fabrication of 3D-Printed Alginate-SNF Scaffold

3D-printed hydrogels based on alginate (5 wt.%) containing various concentrations of SNFs (0, 1, 2, and 3 wt.%) were prepared using a 3D printer. In this regard, SNFs were firstly dispersed in DDW using ultrasonic equipment (WUDD10H, 770W, Korea) for 30 s and then mixed for 2 h at room temperature. It is worth mentioning that the concentration of alginate and SNF solutions were optimized with trial and error according to the concentration of each component in previous studies [35,36]. The preliminary trials aimed to formulate inks having suitable viscoelastic properties so that they flow through the nozzle and retain their structure after being deposited. For instance, clogging was experienced when the concentration of SNF and alginate was more than 3 wt.% and 5 wt.% in 3D printing.

After mixing the 2 above solutions overnight to get a homogenous solution, the inks were printed using the modified FDM 3D printer with a 300-µm nozzle. The models of scaffolds were designed with Catia software (V.5), then sliced with Ultimaker Cura3 to get G-code output usable on the Printer device. Printing parameters were also set to a 3–5 mm·s^−1^ speed rate, flow rate 100–500%, and 0.05–0.07 mm dosing distance. The 3D-printed hydrogels were cross-linked using 90 mM CaCl_2_ by spraying during printing (Figure 1B). The interaction between matrix and additive is also schematically illustrated in Figure 1B. According to the concentration of SNF (0, 1, 2, and 3 wt.%), the samples were named Alg-0SNF, Alg-1SNF, Alg-2SNF, and Alg-3SNF, respectively.

### 2.4. Characterization of Alginate-SNF Hybrid Hydrogel

X-ray diffraction (XRD, X’Pert Pro X-ray diffractometer, Phillips, Germany) and Fourier transform infrared spectroscopy (Tensor27, Bruker, Germany), operating in the wavenumber range of 4000 cm^−1^–400 cm^−1^, were used to characterize alginate, hybrid hydrogels and SNFs. The β-sheet content of degummed SNFs in the nanofibrillar state and after the 3D printing process was estimated using the FTIR spectra and Equation (1) [37]:

(1) $$\beta \text{-}sheet\;content\;\left(\%\right)=\frac{A_{1515}}{A_{1630}+A_{1515}}\times 100$$

where *A*_1515_ and *A*_1630_ represent the areas under the bands at 1515 and 1630 cm^−1^, respectively. These bands are related to amide I and amide II in the structure of silk, respectively. Also, scanning electron microscopy (SEM, Philips, XL30, Eindhoven-Netherland was applied to examine the microstructure of 3D-printed hydrogels. Before imaging, the samples were freeze-dried for 24 h after being submerged in liquid nitrogen and were then gold-coated using a Bal-Tec SCD 050 Sample Sputter Coater (Bal-Tec AG, Switzerland). Furthermore, the pore size distribution of the 3D-printed hydrogels (n = 10) was determined using ImageJ software and SEM images.

The mechanical characteristics of Alg-SNF hydrogels under compressive and tensile situations were evaluated using a tensile tester (Hounsfield H25KS, United Kingdom) with a load cell capacity of 500 N. In the compressive testing, the hydrogels (n = 3) with a diameter × height of 9.75 × 17.5 (mm × mm) were fabricated and then cross-linked using 90 mM CaCl_2_ solution. The samples were compacted with the strain rate of 2 mm.min^−1,^ and then the compressive strength (at strain = 70%) and modulus were calculated using the stress-strain curves. To investigate the tensile behavior of 3D-printed hydrogels, specimens were printed using ISO 527-2/1B/2 parameters and a gauge length of 40 mm. After crosslinking using 90 mM CaCl_2_ solution, the samples were put on a piece of tape in the tension grips of a tensile tester Hounsfield H25KS (United Kingdom) and exposed to the tensile strain with a constant rate of 2 mm/min. The elastic modulus was examined by determining the slope of the stress-strain curves in the linear area. Furthermore, a rheometer (Anton Paar GmbH, Graz, Austria) was also used to measure the viscoelastic characteristics of pre-polymers to show the printability of ink. The test temperature was set at 25 °C.

To investigate the effects of SNF on the rheological properties and printability, rheological testing was performed using an MCR 502 rheometer (Anton Paar, Graz, Austria) equipped with 25 mm parallel plate geometry at a distance of 1 mm. At first, a flow test was performed to assess the viscosity of the polymer solution, while the shear rate was modulated at 0.01–100 s^−1^ at 25 °C. The power law equation (Equation (2)) was also employed to study the shear viscosity of the ink (η) as a function of shear rate (γ) [38]:

η = K · γ^n−1^ (2)

where K and n define the consistency index and flow index, respectively. The exponent (n) was used to determine the flow properties of inks. While n < 1 implies the shear thinning properties, n > 1 proves the shear thickening ability. Moreover, n = 1 shows the Newtonian flow characteristic. An oscillatory frequency sweep was also used at 0.01–100 Hz and 25 °C. The strain was kept constant. From the LVR (linear viscoelastic region), 0.1–1% strain was selected for the oscillation frequency evaluation conducted at a frequency range of 10^−2^–10^2^ Hz.

The effect of SNFs on the dimension changes of scaffolds after cross-linking was examined by immersion of scaffolds in CaCl_2(aq)_ for 10 min and measuring dimensions before and after crosslinking [39]. Swelling and degradation tests were also performed to investigate the role of SNF concentration on the physiological stability of 3D Alg-SNF scaffolds. To measure the swelling ratio, the samples (n = 3) were freeze-dried, weighed (W1), and then immersed in phosphate buffer solution (PBS, pH = 7.4, 37 °C) for 2 h. The hydrogels were weighed (W2) after being wiped off, and the swelling ratio was estimated using Equation (3) [40]:

(3) $$Swelling\;ratio\;\left(\%\right)=\frac{W2-W1}{W1}\;\times 100\;$$

In addition, to investigate degradation properties, the scaffolds (n = 3) were freeze-dried and weighed (*W_I_*). Then, samples were submerged in a PBS solution with a pH of 7.4 for 14 days. At each time point (1, 3, 5, 7, and 14 days), the weight of the freeze-dried samples was recorded (*W_F_*), and the degradation rate was recorded, based on Equation (4) [41]:

(4) $$Degradation\;rate\;\left(\%\right)=\frac{W_{F}-W_{I}\;}{W_{I}}\times 100$$

### 2.5. Cell Culture Investigations

Mouse L929 fibroblast cells (received from Royan institute, Isfahan, Iran) were used to study the cellular behavior of 3D-printed Alg-SNF scaffolds. In this regard, the samples with a dimension of 1 × 1 cm^2^ were sterilized under the UV light for 20 min. Then, the cells were seeded on them and in tissue culture plastic (TCP as control), with a density of 10,000 cells/sample, and incubated in the Dulbecco′s Modified Eagle′s Medium (DMEM, Sigma-Aldrich, St. Louis, USA) enriched with 10% Fetal bovine serum (FBS, Bioidea, Tehran, Iran), 1% Gentamicin (Sigma-Aldrich, Taufkirchen, Germany), and 1% (*v*/*v*) GlutaMax (Bioidea, Tehran, Iran) for 5 days. After 1st and 5th day(s), the calcein-AM/ethidium homodimer (EthD-III) live/dead test (Biotium, UK) was utilized to determine the vitality of cells. The cell-cultured samples were rinsed, and then 100 µL of a live/dead solution containing 2 M calcein AM and 4 M ethidium homodimer was added to cover the samples. After 1 h incubation (n = 3) at 37 °C, the samples were imaged using an inverted fluorescent microscope (Nikon TE2000-U, Japan). Lastly, the Image J program was used to calculate the cell viability by dividing the number of living cells (green cells) by the whole cell number (green + red stains).

MTT test was also done, based on the manufacturer protocol (Sigma), to examine the relative cell growth. The culture media was removed at the specified periods (1, 3, and 5 days) and replaced with MTT solution (5 g·mL^−1^). After 3 h incubation at 37 °C, the formed formazan crystals were dissolved in DMSO (Merck, Darmstadt, Germany), and the optical density (OD) of each solution was measured against DMSO (blank) at the wavelength 490 nm using an ELISA reader (Biotek Instruments, China). The relative cell viability (%) was computed using Equation (5) [42].

(5) $$Relative\;cell\;viability\;\left(\%\right)=\frac{A_{sample}-A_{b}\;}{A_{c}-A_{b}}\times 100$$

where *A_Sample_*, *A_b_*, and *A_c_* represent the absorbance of the sample, blank (DMSO), and control (TCP), respectively.

DAPI/phalloidin staining was performed to investigate the role of SNF on the cytoskeletal structure (F-actin) of fibroblasts. At the specific time points, the cell-seeded samples were fixed using a 4% paraformaldehyde (Sigma-Aldrich, St. Louis, USA) solution for 20 min. After two-time rinsing, the cells were permeabilized in 0.1% Triton X-100 (Sigma-Aldrich, St. Louis, USA) for 5 min. The cells’ actin filaments were stained using a 1:40 dilution of rhodamine phalloidin (Cytoskeleton Inc., Denver, USA) solution for 20 min, and then the cell nuclei were stained using a 1:1000 dilution of 40, 6-diamidino-2-phenyl indole dihydrochloride (DAPI, Sigma-Aldrich, St. Louis, MO, USA) in PBS. Finally, a fluorescence microscope was used to examine the stained samples. Moreover, the cell area of each sample (n = 3) was measured in order to quantify cell density.

### 2.6. Statistical Analysis

A 1-way ANOVA was used to statistically analyze the results. The significant difference between groups was reported according to the Tukey–Kramer post hoc test by GraphPad Prism Software (V.8). *p*-values < 0.05 were treated as statistically significant.

## 3. Results and Discussion

### 3.1. Chemical Characterization of 3D-Printed Alg-SNF Scaffolds

To create a shear-thinning ink and a robust 3D-printed hydrogel for soft tissue engineering, we incorporated SNFs into alginate hydrogel. SNFs extracted from silkworm cocoons were applied to mimic the fibrous proteins of the native extracellular matrix [43]. According to Figure 1C, silk fibroin possesses complex hierarchical structures that combine crystalline and amorphous phases providing beneficial characteristics [44]. SNFs, as the fundamental building blocks of natural silk fibers, not only have the advantages of their natural origin but also may increase the printability of the ink owing to their higher aspect ratio and more functional groups. SEM image in Figure 1A demonstrated the formation of SNFs from silk fibroin with a wide size distribution ranging from nanometer to micrometer sizes. The advantages of our process are its simplicity and the fast nanofibril formation using an aqueous procedure, avoiding the use of any toxic material. FTIR spectrum of SNF in Figure 1D also confirmed the successful fibrillation of silk fibroin. The spectrum of SNFs consisted of amide I (C=O stretching) and amide II (N–H) at 1630 cm^−1^ and 1515 cm^−1^, respectively, confirming the fibroin structure [34]. Three absorption bands were detected at 820 cm^−1^, 890 cm^−1^, and 942 cm^−1^ in the area of primary aliphatic amines (-N_2_H) in the FTIR spectrum related to SNF. FTIR spectrum of alginate also consisted of the distinct bands of COOH (1420 cm^−1^), O-H stretching (1034 cm^−1^), and the asymmetric and symmetric stretching of carboxylate -COO- at 1610 cm^−1^ and 1408 cm^−1^ [45]. After the formation of 3D-printed Alg-SNF hydrogel, the distinct bands of both alginate and SNF were detected, including the band at 1416 cm^−1^ that is attributed to COOH. Moreover, the intensity of the characteristic band of SNF at 1630 cm^−1^ related to C=O was improved, and the characteristic carboxyl groups of alginate were also shifted from 1420 cm^−1^ to 1416 cm^−1^, confirming the interaction between SNF’s amide groups and Alginate’s carboxyl groups based on intermolecular hydrogen bonds, without posing any alteration to the structure [30,46,47]. It has been reported that physical interactions such as hydrogen bonding play significant roles in 3D printing, including endowing the materials with shear responsiveness, enhancing their processability, improving interlayer adhesion and mechanical strength, modulating the viscosity, and providing constructs with self-healing and shape memory properties [48]. Inspired by these studies, the hydrogen bonding between the N-H group of SNF and the COOH group of the alginate could lead to an enhanced shape fidelity. In addition, according to Equation (1), the β-sheet content of SNF was 48 ± 1%, which was comparable with previous studies [49,50,51]. For instance, Farasatkia et al. [48] found that the β-sheet content of SNF synthesized using the acid-salt method was 53 ± 2%. It could be concluded that the synthesis method and the use of acid-salt solvent instead of LiBr did not have a significant effect on the β-sheet content. However, β-sheet content was decreased to 33 ± 2% after hybrid hydrogel formation, indicating that the alginate could also vaguely improve the random coil formation. Similarly, Xue et al. [51] also found that the β-sheet content of SNF decreased with an increase in magnetic content, while the random coil content improved.

XRD pattern of Alg-SNF, compared to that of SNF and Alg (Figure 1E), also confirmed the presence of both components after the 3D printing process. XRD pattern of alginate consisted of the characteristic peaks related to alginate [52]. The XRD pattern of SNF also consisted of a peak at 2θ = 22°, which was similarly reported in previous studies. It could be attributed to the fibroin structure [53]. After the formation of hybrid Alg-SNF, a slight shift to the higher angles was detected, which might be related to the effect of cross-linking process and the interaction between the matrix and the additive. The alginate matrix consists of -COO groups providing hydrogen bonding. It forced chains to come closer, leading to a decreased inter-planar distance and a change in the peak position [54,55].

The Interaction between the alginate matrix and SNFs also controlled the size stability in hydrogels. According to Figure 1F, the ionic crosslinking using Ca^2+^ resulted in dimensional changes in Alg hydrogel. The incorporation of SNF changed the size stability of hydrogel, depending on the SNF content. Noticeably, while the dimension change of pure alginate hydrogel was 48.8 ± 0.6%, it was reduced to 35.2 ± 1.0% after the incorporation of 3 wt.% SNF. The improved dimension size stability could be due to the reduced water plasticization due to the fibroin’s hydrophobic functional groups. As a result, the distance between polymer chains is reduced, leading to less dimensional change before and after the crosslinking process [56,57]. Similarly, Marksted et al. [58] found that the incorporation of CNFs could also affect the dimensional changes of hydrogel matrices. In addition, Aarstad et al. [14] concluded that the syneresis of Alg-CNF reduced compared to the alginate, revealing that gels contracted less after saturation with calcium when CNFs were added to Alg hydrogel.

### 3.2. Rheological Behavior of 3D-Printed Alg-SNF Scaffolds

**Figure 2:**
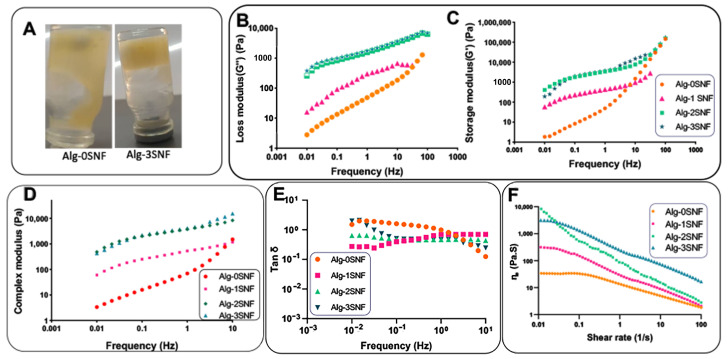
Rheological properties of Alg-SNF formulations: (**A**) The effect of SNF incorporation on the viscoelastic behavior of alginate. (**B**) Loss modulus (G″), (**C**) storage modulus (G′) and (**D**) complex modulus of the ink formulations as a function of frequency. (**E**) Tan δ of the ink formulations. (**F**) The viscosity changes of Alg-SNF inks as a function of shear rate.

The strong interaction between the carboxyl of alginate and amide groups of SNFs also significantly controlled the viscosity of the solution leading to improved stability of hydrogels before crosslinking (Figure 2A). It needs to mention that the inks of extrusion-based printing should have flow and shape retention characteristics. Inks should flow through nozzles with minimal internal resistance. After the material has been distributed, the properties should be reversed, with urgent flow discontinuation, accumulation of internal forces opposing deformation, and elastic shape retention. The ability to show viscous flow and elastic shape retaining is identified as viscoelasticity. [7,59]. We examined the viscoelastic behavior of the hybrid hydrogels. The dynamic elastic modulus (G′) and loss modulus (G″) of all hydrogels at different frequencies are provided in Figure 2B,C. Results indicated all samples had potential printability and indicated a dominance of elasticity since the storage modulus was superior to the loss modulus (G′ > G″), especially at higher frequencies. The elastic-dominated trait (G″ < G′) at a low-frequency sweep could result in a rigid construction after printing [60]. However, the G′ and G″ were the function of the angular frequency (Hz), depending on the sample type. While Alg-0SNF and Alg-1SNF showed a liquid-like behavior, especially at a low frequency, both G′ and G″ were significantly enhanced with the angular frequency at higher SNF content samples. At a high frequency (100 s^−1^), Alg-2SNF and Alg-3SNF revealed a noticeable elasticity or more rigid-like construction after the crossover moduli. The increased modulus of these samples might be due to the structural entanglement, which could be originated from the strong interconnecting networks between carboxylic groups of alginate and amide groups of SNF. Markstedt et al. [15] reported a similar trend for the Alg-CNF hydrogel. The complex modulus (G′ + *i*G″) demonstrated in Figure 2D reveals that the addition of SNFs resulted in a significant improvement in the elastic and loss moduli of alginate, leading to an improved complex modulus. This behavior could be helpful for the printability of hydrogels since polymer solution acts similarly to a viscous material, and the printed material supported its shape. However, the storage modulus of Alg-3SNF was lower than Alg-2SNF in lower frequencies. It might be due to the agglomeration of SNF in Alg-3SNF, which was not conducive to stress transfer, and thus resulting in a decrease in the elastic modulus. Similarly, Jiang et al. [61] reported that agglomeration of CNFs could lead to modulus reduction. The tan δ value (Figure 2E (was also calculated from G″/G′ to evaluate how gel-like inks were. Tan δ values below 1 at the measured frequencies indicate that the inks are more gel-like than liquid [40]. The results showed that the samples became more elastic as the SNF concentration increased and were elastically dominated (tan δ < 1) over the frequency range. However, it was indicated that Alg-0SNF had tan δ > 1 in lower frequencies meaning the viscous behavior in the relaxing time.

According to Figure 2F, the incorporation of SNF also altered the viscosity of the solution. It might be related to the electrostatic interactions between the N-H group of SNF and the -COOH group of the alginate matrix. Our results demonstrated that Alg-SNF hydrogels had greater viscosity than Alg-0 SNF, and the viscosity of the hybrid hydrogels decreased with the shear rate, demonstrating the viscoelastic property of Alg-SNF hydrogels. Other studies similarly found that the electrostatic interactions between the functional groups available in the inks may lead to enhanced elastomeric behavior [62]. In addition, Figure 2F revealed that the viscosity reduced with the increasing shear rate for both Alg and Alg-SNF, confirming shear-thinning behavior. However, the incorporation of SNF in Alg solutions (especially Alg-2SNF and Alg-3SNF) dramatically improved its shear-thinning behavior. For instance, when the shear rate increased from 10^−2^ to 10^2^ s^−1^, the viscosity of Alg-2SNF decreased from 8.2 × 10^3^ to 2.8 × 10^0^ Pa.s. Nevertheless, the viscosity of Alg-0SNF only decreased from 3.4 × 10^1^ to 1.8 × 10^0^ Pa.s in this shear rate range. It could be realized samples with higher SNF concentrations exposed to stress could restore their first shape after stress release. Compared to Alg-CNF [15], Alg-2SNF represented enhanced shear-thinning behavior with the same change in the viscosity observed in a lower range of shear stress. Data regarding the experimental results were also fitted into Equation (2). The (n − 1) values were estimated at about −0.35, −0.571, −0.842, and −0.594 for Alg-0, 1, 2, and 3 SNF samples, respectively. Accordingly, the exponent n proposed that the SNF content considerably affected the shear-thinning behavior of the inks. In addition, it is worth mentioning that Alg-2SNF had better shear-thinning behavior than other inks.

### 3.3. Structural Properties of 3D-Printed Alg-SNF Scaffolds

**Figure 3:**
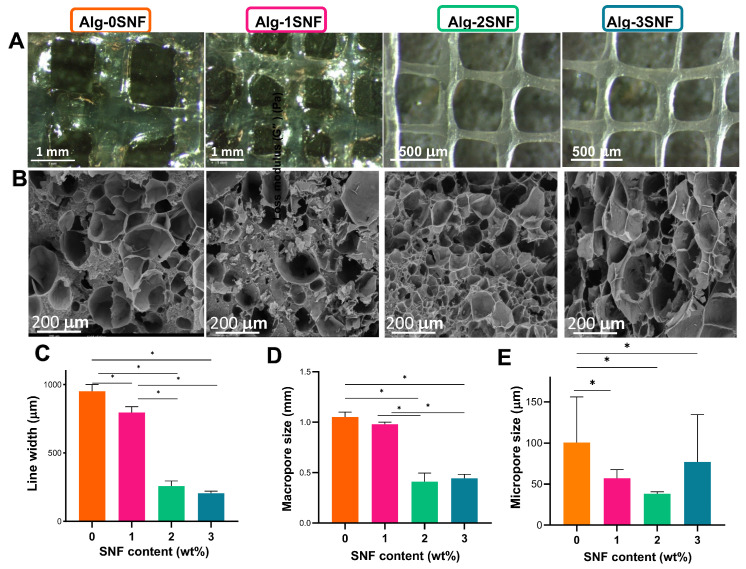
Structural properties of 3D-printed Alg-SNF hydrogels: (**A**) Optical images and (**B**) SEM images of the 3D-printed hydrogels containing various SNF contents. (**C**) Average line width, (**D**) average macro-pore size, and (**E**) average micro-pore size of 3D-printed hydrogels. Data are presented as the mean ± SD (n = 4) (*: Significant differences, * *p* < 0.05.).

To increase the shape fidelity of the alginate, SNF was incorporated as the filler in ink compositions. The effect of SNF on the morphology and micro- and macro-porous structure of 3D-printed alginate hydrogel was studied (Figure 3). Figure 3A,B represents the optical and SEM images of the five-layer grid pattern of hybrid hydrogels, respectively. Comparing small grids printed with Alg-0SNF and Alg-2, three SNF demonstrated how the low viscosity of alginate limited the printing resolution. For instance, according to Figure 3C,D, the strut width of the printed grid decreased, while the macro pore size increased after the incorporation of SNFs. Noticeably, the average strut width of Alg-0SNF was 952 ± 40 μm, which was significantly reduced to 205 ± 14 μm at Alg-3SNF, confirming the viscosity characterization presented in Figure 2F. The increase in the viscosity of inks enhanced the stability of the extruded strands before cross-linking. In addition, SNFs could significantly control the microporous structure of alginate scaffolds. According to Figure 3B, all hydrogels discovered a highly porous network with interconnected porosity. The presence of silk nanofibrils could be identified within the alginate matrix, especially at high SNF content samples (Alg-3SNF). These interconnected porous and fibrous networks could mimic natural ECM matrix and increase the transportation of nutrients and waste products, enabling effective cell functions [63]. However, the pore size and uniformity of hydrogels were significantly changed depending on the hydrogel composition. According to Figure 3E, the average pore size of Alg (100 ± 80 μm) was reduced to 38 ± 2 μm after the incorporation of 2 wt.% SNF (Alg-2SNF) and was then enhanced to 77 ± 50 μm at Alg-3SNF. Anguiano et al. [64] also similarly found a decrease in the pore size of hydroxypropyl cellulose hydrogels after the incorporation of molybdenum disulfide. The reduced pore size of hydrogels could be related to the interaction between alginate and SNF. Interactions between amide and carboxylic groups of the resulting hydrogels led to a much denser and more compact structure, leading to smaller pores [65]. However, the pore size of Alg-3SNF was significantly enhanced compared to other samples. During the freeze-drying step, ice crystals were nucleated and pushed alginate and SNF into interstitial portions of ice crystals. In the Alg-3SNF sample, SNF became conspicuous, and the viscous forces of solutes reduced, so creating bigger ice crystals to develop larger pores [66,67]. In addition, SNF agglomeration and less interaction with the matrix may also result in the non-uniform distribution of pores.

**Figure 4:**
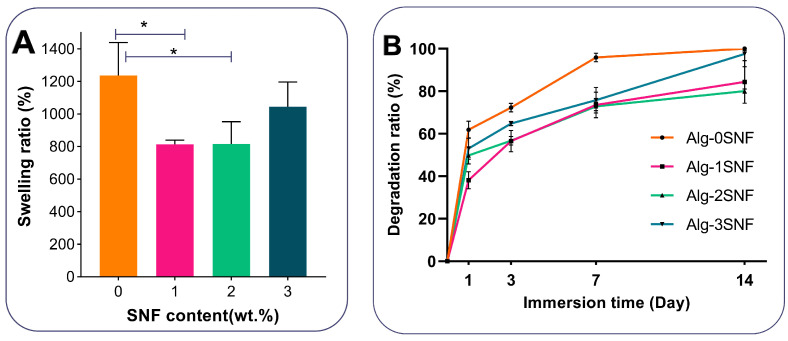
Physiological stability of hybrid Alg-SNF hydrogels: (**A**) The swelling ratio of hydrogels after 1h of soaking in PBS solution at 37 °C. Data are presented as the mean ± SD (n = 3). (*: Significant differences, * *p* < 0.05). (**B**) The weight loss of hydrogels during soaking in PBS for 2 weeks.

One of the benefits of employing hydrogels in tissue engineering is their capacity to absorb water and degrade in biological environments. The swelling behavior of different hydrogel compositions was monitored after 1 h, keeping in PBS at 37 °C. According to Figure 4A, while all hydrogels showed significant swelling ability, SNF could modulate the swelling ratio of the scaffolds. Among various samples, Alg-0SNF showed the maximum swelling ratio of 1273 ± 230% owing to the superior water preservation ability of polysaccharides. During the first times of the swelling process, water was absorbed by capillaries that were presented in the alginate hydrogel. At this condition, the hydrophilic groups (-OH/-COOH) were combined with water molecules to create a hydration layer. However, this value was significantly reduced to 814 ± 35% after the incorporation of 1 wt.% SNF. It could be due to the robust interactions between the available hydrophilic groups of the alginate matrix and SNF. It needs to be mentioned that while both the SNF and Alginate have hydrophilic groups on their molecular chains, SNF is a relatively hydrophobic protein that may decrease the swelling ability. However, the swelling ratio increased to 1046 ± 80% when SNF concentration enhanced to 3 wt.%. It might be due to the agglomeration of SNF at Alg-3SNF, which enhanced the available hydrophilic side chains of alginate for water interaction [68].

The degradation rate of hybrid Alg-SNF hydrogels was also evaluated in PBS (pH = 7.4) for 14 days (Figure 4B). Results showed that the incorporation of 2 wt.% SNF within the alginate hydrogel significantly decreased the weight loss and improved physiological stability, while the swelling ratio was still sufficient for cellular behavior. It might be due to the less swelling ability of hydrogels with increasing SNF content. However, the degradation rate of Alg-3SNF hydrogel was significantly enhanced due to the agglomeration of SNF and its heterogeneous structure. Gharasoo et al. [69] suggested a model confirming that pore-scale heterogeneity could consistently promote degradation rate. According to previous studies, the degradation rate of 3D-printed alginate is not in an appropriate range for various tissue engineering applications, such as cartilage. In this study, alginate and SNF were combined at different ratios to provide finer control over the rate of bioink degradation. Our results demonstrated that the degradation rate of Alginate was significantly reduced after the incorporation of 2 wt.% SNF. This degradation rate was comparable with the results of other 3D-printed scaffolds proposed for cartilage tissue engineering [70], making it promising for this application.

### 3.4. Mechanical Properties of 3D-Printed Alg-SNF Scaffolds

**Figure 5:**
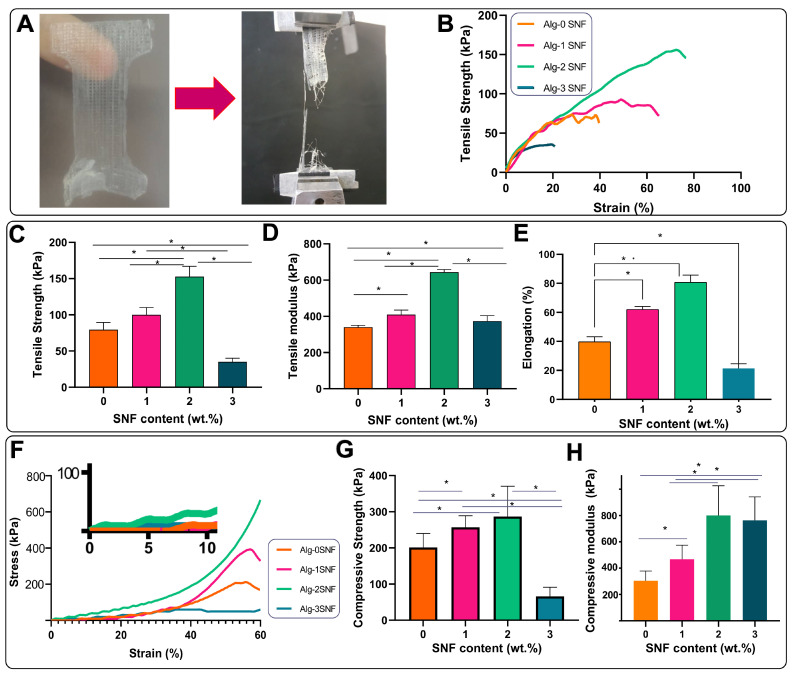
Mechanical properties of Alg-SNF hydrogels: (**A**) Tensile testing of 3D-printed Alg-SNF hydrogels before and after breaking in the stretch mode. (**B**) The representative tensile stress-strain curves of Alg-SNF hydrogels. (**C**) Tensile strength, (**D**) elastic modulus, and (**E**) elongation of Alg-SNF hydrogels. (**F**) The representative compression stress-strain curves of Alg-SNF hydrogels. (**G**) The compressive strength and (**H**) The compressive modulus of Alg-SNF hydrogels. Data are presented as the mean ± SD (n = 5) (*: Significant differences, * *p* < 0.05).

One of the main issues associated with alginate hydrogels is their poor mechanical performance. Here, we investigated the role of SNF on the tensile and compression properties of alginate hydrogel. Figure 5A shows a tensile specimen in a custom-designed gripper adapter. The formation of an aligned broken strut after the tensile test in Figure 5A confirmed that SNF randomly distributed in the hydrogel matrix was aligned with the tension direction. This behavior could result in the improvement of the tensile performance of Alg-SNF hydrogels. The representative stress-strain curves of 3D-printed hydrogels in Figure 5B presented a linear trend until 10% strain, followed by a non-linear behavior. The slope of the linear section was used to determine Young’s modulus [71]. According to these curves, the average tensile strength (Figure 5C), elastic (Young’s) modulus (Figure 5D), and elongation (Figure 5E) were estimated. According to these values, it could be concluded that the SNF content had a significant effect on the tensile performances of the hybrid hydrogels. The tensile strength of alginate hydrogel was significantly enhanced (2 times) with increasing SNF content up to 2 wt.% (*p* < 0.05). Moreover, incorporation of SNF up to 2 wt.% significantly enhanced (about 2 times) the elastic modulus from 324 ± 8 kPa (in Alg-0SNF) to 643 ± 15 kPa (*p* < 0.05) and then reduced with increasing SNF content up to 3 wt.%. This behavior probably originated from the hydrogen bonding between Alg and SNF, which led to the creation of a stiffer hydrogel matrix [72]. It needs to mention that the tensile behavior of alginate hydrogels is highly dependent on alginate type, formulation, gelling conditions, incubation, and strain rate [73]. However, the improvement of tensile performances after the incorporation of SNF was similarly reported in previous studies. Liling et al. [74] and Barros et al. [75] demonstrated that the mechanical properties of sodium alginate hydrogels were mainly influenced by the type and concentration of the cross-linker agents and the presence of additives. In addition, the tensile performances of silk-incorporated scaffolds are influenced by β-sheet content. β-sheet crystals in SNFs created using strong supramolecular interactions and afforded mechanical stiffness and stable features. When SNFs are bared tensile forces, β-sheet nanocrystals and chains could partially orient by the creation of interlocking parts transferring the load between chains. However, the incorporation of 3% SNF significantly reduced the mechanical strength and elongation. Generally, the mechanical properties of hybrid hydrogels strongly depend on not only the intrinsic characteristics of reinforcements but also the good distribution of these reinforcements. Therefore, the poor dispersion and aggregation of SNFs in the alginate matrix was a significant barrier to the formation of uniform Alg-3SNF leading to the formation of a heterogeneous structure containing large pores with large size distribution [49]. A similar result was reported for silk fibroin and methacrylate gelatin (GelMA) hydrogels [76,77]. It was found that the incorporation of CNFs within the fibroin matrix significantly improved tensile performances, owing to the strong interaction between SF protein and CNFs.

The compressive stress-strain curves were provided, up to 60% strain (Figure 5F). The stress-strain curves at 0–10% strain were also presented with higher magnification. Depending on the SNF content, the hybrid hydrogels were destroyed in 70–90%. Our results revealed while the pure alginate sample failed at about 70%, all hybrid hydrogels could bear more than 90% strain before failure. Moreover, the compressive strength of hybrid hydrogels was significantly changed depending on the SNF content. It was also found that there was no significant difference between the compressive stress of the constructs at lower strains (<20%), followed by an increase in stress. In the first linear section, during the stress application, the hydrogel experienced a stressed status and started to an elastic deformation to preserve energy and repel the compressive stress. This deformation might be related to the free water loosening, which was not wholly captured in the hydrogel matrix. The considerably improved stress after 20% strain might be related to the reaching deformation to its limit value, making the following deformation more difficult [39]. For example, at 50% strain, the compressive strength enhanced from 201 ± 42 kPa (for the Alg-0SNF) to 286 ± 83 kPa (for the Alg-2SNF) (Figure 5G). This trend was similarly observed in compressive modulus. According to Figure 5H, the incorporation of SNF significantly enhanced the compressive modulus and ranged from 305 ± 50 kPa at Alg-0SNF to 800 ± 170 kPa at the Alg-2SNF sample. The exfoliated SNF displayed a fibrous structure, and if uniformly dispersed in an aqueous solution, according to the interaction between SNF and alginate, SNFs could transfer the stress in the alginate matrix [39]. Our results revealed the uniform distribution of SNFs could efficiently reinforce the alginate scaffold, which might be related to the high mechanical performances of SNFs and the strong interfacial interactions between the SNF and alginate. This behavior was similarly reported in other biopolymer nanofibrils-reinforced hydrogels [78,79]. Another parameter controlling the compressive performances of the hydrogel was pore size (Figure 3E). The strength of hydrogels reduced with the increase in pore size in agreement with swelling data. This behavior was similarly reported in previous studies [49]. Compared to similar studies, the compressive behavior of Alg-SNF hydrogels was more significant. For instance, the compressive modulus of optimized Alginate-CNFs was reported to be about 230 KPa [39]. Generally, the stiffness of a material plays a critical role as it affects the cell-material interaction [80]. Handorf et al. [81] found that the stiffness of the ECM affects cell proliferation, the direction of cell migration, cell adhesion and cell differentiation. According to Thiele et al. [82], soft matrices (0.1–1 kPa) are neurogenic and, therefore, mimic brain tissue. Stiffer matrices (8–17 kPa) are myogenic and mimic muscle tissue, while rigid matrices (25–40 kPa) are osteogenic and, therefore, mimic collagenous bone. Due to the differences in microstructure between the different cartilage zones [83], the compression modulus varies between 230 kPa–790 kPa [84]. In this study, it was found that Alg-SNF hydrogels with stiffness in the range of 130–346 kPa could be appropriate for cartilage tissue engineered.

### 3.5. Cell Culture Investigation

**Figure 6:**
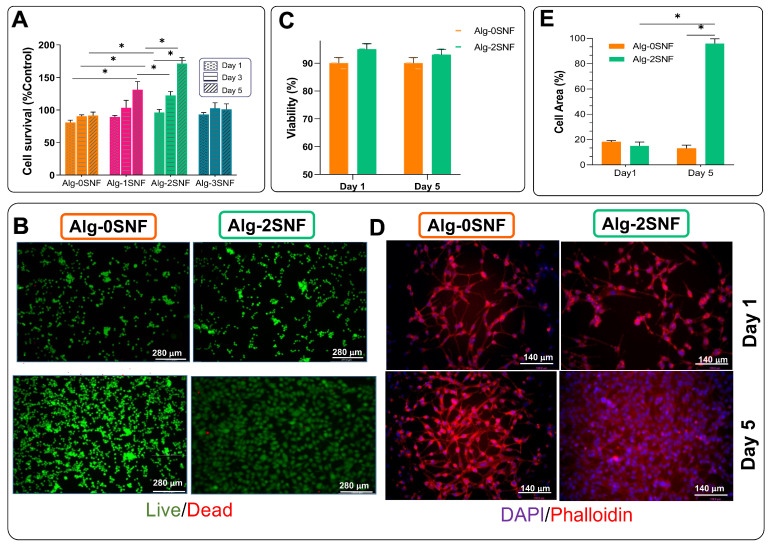
Biological properties of Alg-SNF hydrogels: (**A**) Normalized survival of fibroblasts cultured on Alg-SNF determined using MTT assays during 5 days of culture; (**B**) Representative fluorescence images; (**C**) quantified cell viability determined using live/dead assay after 1 and 5 days of culture. The cells were stained with Calcein AM (green) and EthDII (red) exhibiting live and dead cells, respectively; (**D**) Fluorescence images; and (**E**) the spreading, the fraction of area covered with cell clusters, of fibroblasts after 5 days of culture. The actin cytoskeleton and nuclei of cells were stained with rhodamine-phalloidin (red) and DAPI (blue), respectively. Data are presented as the mean ± SD (n = 3) (*: Significant differences, * *p* < 0.05.).

To study the role of SNF on the biological properties of the 3D-printed alginate hydrogel, fibroblasts were seeded on the samples. According to Figure 6A, a substantial increase in cell survival after 5 days of culture was detected, confirming all samples were cytocompatible. Moreover, the addition of SNF to alginate hydrogel resulted in a 1.7-fold increase in cell survival (from 91 ± 5% control (for Alg-0SNF) to 168 ± 9% (for Alg-2SNF)). However, the incorporation of more SNF content up to 3 wt.% decreased the cell survival, which might be due to the enhanced degradation rate and lack of sufficient mechanical properties [85]. The improved cell viability after the incorporation of SNFs was similarly reported in previous studies. For instance, Nikam et al. [86] reported cell adhesion to the silk nanofibril was significantly enhanced. According to the optimized shear thinning, mechanical properties, and physiological stability of Alg-2SNF, this sample was selected for further biocompatibility evaluation.

To study the cell viability in contact with Alg-2SNF and Alg-0SNF, a Live/Dead assay was performed. Figure 6B shows the representative fluorescence microscopy images of cells seeded on the samples, showing both live (green) and dead (red) cells cultured for 1 and 5 days. According to Figure 6C, while cell viability was more than 90%, the density of live cells significantly enhanced in contact with the Alg-2SNF sample. The role of nanofibrils in enhancing cell adhesion and viability was confirmed in the literature [87]. The improved cell viability and proliferation could be related to nanofibrous architecture providing anchor sits for the cells. Nanofibrous scaffolds improved the adsorption of some types of proteins, including fibronectin, vitronectin, and laminin, admitting the cells to anchor more tightly to the matrix. Consequently, it could result in a higher number of cells attached [88].

**Figure 7:**
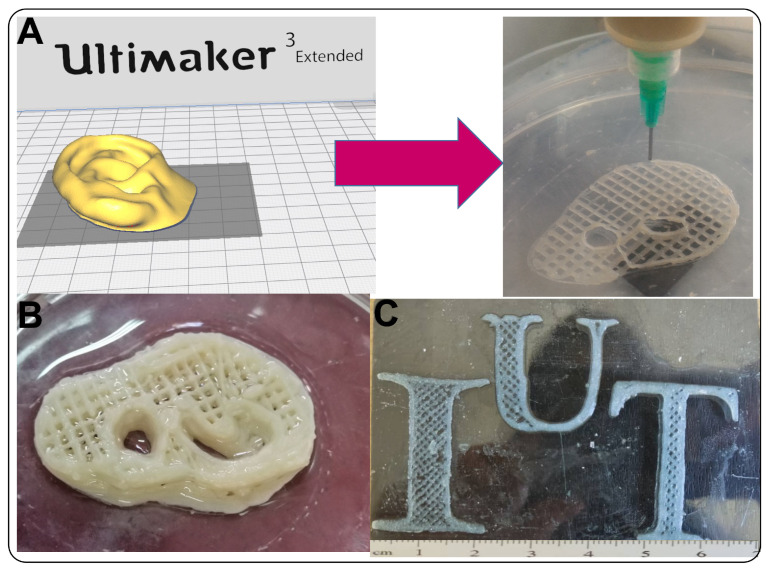
Alg-SNF bioinks for printing complex structures: (**A**) A 3D model of ear shape in software and 3D printing of the right ear using Alg-SNF hydrogel. (**B**) Alginate-SNF ear-shaped hydrogel. (**C**) IUT logo printed with Alg-SNF ink showing the ability of ink to print pre-designed models.

To evaluate the role of 3D-printed hydrogel composition on the cell spreading, the F-actin and nuclei were stained (Figure 6D), and the cell area on various hydrogels was measured (Figure 6E). Our results showed that while the fraction of surface hydrogel covered with fibroblasts after a day of culture was similar to fibrillated and pure alginate, the fraction of hydrogels covered with fibroblasts significantly improved from 51.4 ± 4.1% (at pure Alginate) to 96.1 ± 3.2% (at Alg-2 SNF), showing the effective role of SNF on the improved cell proliferation and spreading. Generally, the cell fate could be controlled using various chemical and physical parameters of the microenvironment, called cell niche [85,89]. Significantly, the mechanical properties of the cell matrix, such as the stiffness, can act as powerful signals for the control of cell functions, including cell proliferation, migration, and differentiation [90,91]. Our results showed that the incorporation of SNF within alginate hydrogel resulted in the stimulation of the natural ECM and improved mechanical properties leading to enhanced cell adhesion and spreading. Our results demonstrated that Alg-2SNF bioink with significant shear thinning behavior could be promising for developing a 3D-printed scaffold with desired mechanical properties, physiological stability, and biological properties. Alg-2SNF bioink was also successfully used to create 3D-printed complex shapes resembling ear cartilage and the IUT logo (Figure 7B,C). The ability to easily control the pore size and layer numbers using Cura parameters in contrast to other studies [92] are attractive properties of this ink making it appropriate for 3D printing complex tissues.

Generally, the biocompatibility of tissue substitutes is essential in order to exclude short- and long-term health impairment. Furthermore, there is growing evidence that mechanical properties are fundamental for cellular behavior and consequent tissue functionality [24]. According to the intrinsic properties of SNF, it could mimic the structural and, subsequently, mechanical behavior of functional tissues such as tendons, ligaments, and menisci, having immense strength and stiffness [24]. By modifying the nanofibril volume fraction in the alginate matrix, the mechanical performances of Alg-SNF were in the near range of native human soft tissues such as auricular cartilage [93]. Therefore, 3D-printed Alg-SNF hydrogel could have the potential to be used for cartilage tissue engineering.

## 4. Conclusions

In this study, a 3D-printed hydrogel based on alginate-silk nanofibril (Alg-SNF) was introduced for soft tissue engineering. SNF significantly changed the injectability of alginate by improving its shear-thinning behavior and shape retention before ionic crosslinking. Furthermore, the rheological and mechanical properties, as well as the physiological stability of Alg-SNF hydrogels, were significantly modulated depending on the SNF content. Noticeably, the incorporation of 2 wt.% SNF significantly enhanced tensile strength (5 times) and compressive strength (3 times) while reducing degradation rate (1.6 times after 14 days of incubation) and swelling ratio (1.5 times), compared to 3D-printed alginate hydrogel. Moreover, 3D-printed Alg-SNF hydrogel could maintain the attachment and proliferation of cells in vitro. Finally, hybrid Alg-SNF hydrogel with desired physical and mechanical properties could be 3D-printed in complex shapes such as ear cartilage. Taken together, our results proposed the potential of Alg-SNF ink for the engineering of soft tissues.

## Data Availability

The data presented in this study are available on request from the corresponding author.
